# Cancer Genome Interpreter annotates the biological and clinical relevance of tumor alterations

**DOI:** 10.1186/s13073-018-0531-8

**Published:** 2018-03-28

**Authors:** David Tamborero, Carlota Rubio-Perez, Jordi Deu-Pons, Michael P. Schroeder, Ana Vivancos, Ana Rovira, Ignasi Tusquets, Joan Albanell, Jordi Rodon, Josep Tabernero, Carmen de Torres, Rodrigo Dienstmann, Abel Gonzalez-Perez, Nuria Lopez-Bigas

**Affiliations:** 1 Research Program on Biomedical Informatics (GRIB), IMIM Hospital del Mar Medical, Research Institute and Pompeu Fabra University, Barcelona, Spain; 2 Institute for Research in Biomedicine (IRB Barcelona), The Barcelona Institute of Science and Technology, Barcelona, Spain; 3 Charité – Universitätsmedizin Berlin, Berlin, Germany; 4 Vall d’Hebron Institute of Oncology, Vall d’Hebron University Hospital, Autonomous University of Barcelona, Barcelona, Spain; 5 Hospital del Mar Medical Research Institute (IMIM), Barcelona, Spain; 6 Department of Oncology, Hospital del Mar- CIBERONC, Barcelona, Spain; 7 Autonomous University of Barcelona, Barcelona, Spain; 8 Pompeu Fabra University, Barcelona, Spain; 9 Developmental Tumor Biology Laboratory, Institut de Recerca Pediàtrica- Hospital Sant Joan de Déu Barcelona, Barcelona, Spain; 10 Institució Catalana de Recerca i Estudis Avançats (ICREA), Barcelona, Spain

## Abstract

While tumor genome sequencing has become widely available in clinical and research settings, the interpretation of tumor somatic variants remains an important bottleneck. Here we present the Cancer Genome Interpreter, a versatile platform that automates the interpretation of newly sequenced cancer genomes, annotating the potential of alterations detected in tumors to act as drivers and their possible effect on treatment response. The results are organized in different levels of evidence according to current knowledge, which we envision can support a broad range of oncology use cases. The resource is publicly available at http://www.cancergenomeinterpreter.org.

**Electronic supplementary material:**

The online version of this article (10.1186/s13073-018-0531-8) contains supplementary material, which is available to authorized users.

## Background

The accumulation of so-called “driver” genomic alterations confers on cells tumorigenic capabilities [1]. Thousands of tumor genomes are sequenced around the world every year for both research and clinical purposes. In some cases the whole genome is sequenced while in others the focus is on the exome or a panel of selected genomic regions. It then becomes necessary to annotate which of the somatic mutations identified by the sequencing have a possible role in tumorigenesis and treatment response. This process, which we refer to as “the interpretation of cancer genomes”, is currently tedious and largely unsolved. One of its major bottlenecks is the identification of the alterations driving the tumor. A widely employed approach to solve this hurdle consists in focusing on the mutations affecting known cancer genes, i.e., tumor suppressors and oncogenes. These were initially identified through experimentation, giving rise over the past 40 years to a stable census of human cancer genes [2]. More recently, projects re-sequencing large cohorts of tumors have provided the opportunity to systematically identify genes involved in tumorigenesis through the detection of signals of positive selection in their alteration patterns across tumors of some two dozen malignancies [3–6]. However, many of the somatic variants detected in tumors, even those in cancer genes, still have uncertain significance and thus it is not clear whether or not they are relevant for tumorigenesis. Another hurdle in the interpretation of cancer genomes concerns one of its crucial aims: the identification of tumor alterations that may affect treatment options. Unstructured information on the effectiveness of therapies targeting specific cancer drivers is continuously generated by clinical trials and pre-clinical experiments, and currently several resources are dedicated to gather and curate these data, such as ClinVar [7], DoCM [8], OncoKB [9], My Cancer Genome (https://www.mycancergenome.org), PMKB [10], PCT (https://pct.mdanderson.org), CIViC [11], and JAX-CKB (https://ckb.jax.org). Nevertheless, none of these resources address the whole process of interpretation and researchers and clinicians thus face a challenging task to annotate the variants detected in a newly sequenced cancer genome with their collective information.

Here, we describe the Cancer Genome Interpreter (CGI), a platform that systematizes the interpretation of cancer genomes, the main hallmark of which is the streamlining and automatization of the whole process (Additional file 1: Table S1). Specifically, the CGI addresses the two aforementioned challenges. On the one hand, it identifies all known and likely tumorigenic genomic alterations (point mutations, small insertions/deletions, copy number alterations and/or gene fusions) of a newly sequenced tumor, including the assessment of variants of unknown significance. On the other, it annotates all variants of the tumor that constitute state-of-the-art biomarkers of drug response organized using different clinical evidence. The CGI accepts several data formats and its output reports are provided in a user-friendly interactive framework that organizes the results according to distinct levels of clinical relevance, which may thus be used in a broad range of applications.

## Construction and content

**Fig. 1:**
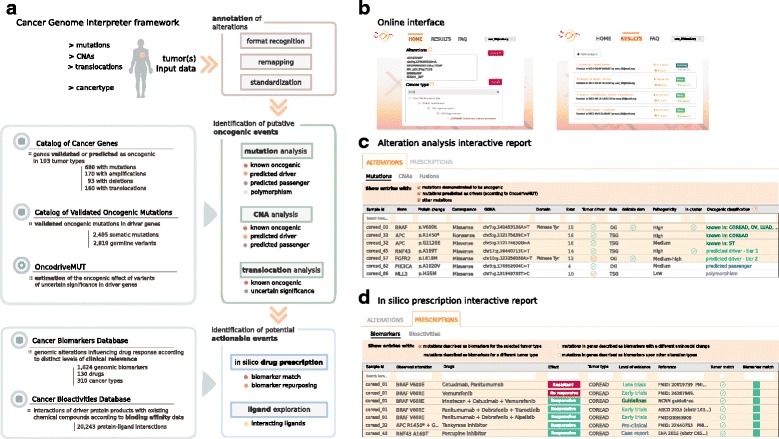
Cancer Genome Interpreter. **a** Outline of the CGI workflow. With a list of genomic alterations as input, the CGI automatically recognizes the format, remaps the variants as needed, and standardizes the annotation for downstream compatibility. All analyses are cancer-specific and thus the tumor type of the sample(s) to analyze is also required. Next, the CGI identifies known driver alterations and annotates and classifies the remaining variants of unknown significance. Finally, alterations that are biomarkers of drug effects are identified. **b** The CGI may be run via the web at http://www.cancergenomeinterpreter.org (*left panel*) or through an API. The web results can be stored in a private repository (*right panel*) for their management. The results of the CGI are provided via interactive reports. **c** An example of a mutation analysis report. This contains the annotations of all mutations, which empowers the user’s review, and the labels for those known or predicted to be drivers by OncodriveMUT. **d** An example of a biomarker match report. This contains the putative biomarkers of drug response found in the tumor organized according to distinct levels of clinical relevance. All these web reports are interactive and configurable by the user. *CNA* copy number alteration

The CGI employs existing or newly developed resources and computational methods to annotate and analyze the alterations in a tumor according to distinct levels of evidence (Fig. 1a; details in Additional file 2: Note I). The tool is freely available through an API or a web interface at http://www.cancergenomeinterpreter.org, under an open license, with the aim of facilitating its use by cancer researchers and clinical oncologists (Fig. 1b–d). In the following sections we present the blueprint for the interpretation of cancer genomes implemented by the CGI, describe the resource, and discuss its utility.

### A comprehensive catalog of cancer genes across tumor types

**Fig. 2:**
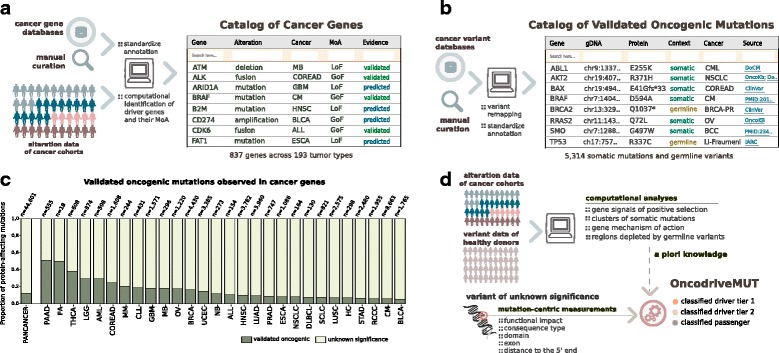
Annotating mutations in cancer genes. **a** Catalog of Cancer Genes. Genes that drive tumorigenesis via mutations, copy number alterations, and/or translocations are annotated with their mode of action (*MoA*). **b** Catalog of Validated Oncogenic Mutations. Clinically or experimentally validated driver mutations were gathered from manually annotated resources and the cancer literature. **c** Proportion of validated mutations observed across the cancer genes of 6792 tumors. Cancer types: *ALL* acute lymphocytic leukemia, *AML* acute myeloid leukemia, *BLCA* bladder carcinoma, *BRCA* breast carcinoma, *CLL* chronic lymphocytic leukemia, *CM* cutaneous melanoma, *COREAD* colorectal adenocarcinoma, *DLBC* diffuse large B cell lymphoma, *ESCA* esophageal carcinoma, *GBM* glioblastoma multiforme, *HC* hepatocarcinoma, *HNSC* head and neck squamous cell carcinoma, *LGG* lower grade glioma, *LUAD* lung adenocarcinoma, *LUSC* lung squamous cell carcinoma, *MB* medulloblastoma, *MM* multiple myeloma, *NB* neuroblastoma, *NSCLC* non-small cell lung carcinoma, *OV* serous ovarian adenocarcinoma, *PA* pilocytic astrocytoma, *PAAD* pancreas adenocarcinoma, *PRAD* prostate adenocarcinoma, *RCC* renal clear cell carcinoma, *SCLC* small cell lung carcinoma, *STAD* stomach adenocarcinoma, *THCA* thyroid carcinoma, *UCEC* uterine corpus endometrioid carcinoma. **d** OncodriveMUT schema to estimate the oncogenic potential of the variants of unknown significance. A set of heuristic rules combines the annotations obtained for a given mutation with the knowledge about the genes (or regions thereof) in which it is observed, as retrieved from the computational analyses of sequenced cohorts

One of the main aims of the interpretation of cancer genomes is to identify the alterations responsible for tumorigenic traits. In the CGI, this process begins with a focus on alterations that affect the genes capable of driving the cancer hallmarks of a particular tumor type. Therefore, we compiled a catalog of genes involved in the onset and progression of different types of cancer, obtained via different methods and from different sources (Additional file 2: Note II). First, from manually curated resources [2, 7, 8, 12, 13] and the literature we collected genes that have been experimentally or clinically verified to drive tumorigenesis. Second, we incorporated the results of bioinformatics analyses of large tumor cohorts re-sequenced by international initiatives such as The Cancer Genome Atlas (http://cancergenome.nih.gov/abouttcga) and the International Cancer Genome Consortium [14] (specifically, we identified genes whose somatic alterations exhibit signals of positive selection across 6729 tumors representing 28 types of cancer [4]). Each of these cancer genes was annotated with their mode of action in tumorigenesis (i.e., whether they function as oncogenes or tumor suppressors), on the basis of either experimentally verified sources, or *in silico* prediction [15]. The resulting Catalog of Cancer Genes currently comprises 837 genes with evidence of a tumorigenic role in 193 different cancer types (Fig. 2a). Each entry in the catalog thus includes, along with the name of the driver gene, (i) the malignancies it drives, organized according to available evidence; (ii) the types of alterations involved (mutations, copy number alterations, and/or gene translocations); (iii) the source(s) of this information; (iv) the context (germline or somatic) in which these alterations are tumorigenic; and (v) the gene’s mode of action in cancer as appropriate. The catalog is available for download through the CGI website (https://www.cancergenomeinterpreter.org/genes).

### Most mutations affecting cancer genes are of uncertain significance

The focus on cancer genes described above is a necessary but not sufficient to identify the tumorigenic variants in a tumor, since not all variants observed in a cancer gene are necessarily capable of driving tumorigenesis. Therefore, the CGI next focuses on annotating and analyzing protein-affecting mutations (PAMs) that occur in genes of the Catalog of Cancer Genes. First, validated tumorigenic mutations may confidently be labeled as drivers when detected in a newly sequenced tumor. We compiled an inventory that currently contains 5314 such validated mutations, including cancer-predisposing variants, from dedicated resources [7–9, 12, 13, 16] and the literature (Fig. 2b; Additional file 2: Note III). This Catalog of Validated Oncogenic Mutations is available for download through the CGI website (https://www.cancergenomeinterpreter.org/mutations). Across a pan-cancer cohort of 6792 tumors sequenced at the whole-exome level (mostly at diagnosis) [4] we observed that only 5360 (916 unique variants) of the 44,601 PAMs found in cancer genes appear in this catalog. In other words, 88% of all PAMs that affect cancer genes in this cohort are currently of uncertain significance for tumorigenesis, a proportion that varies widely per gene and tumor type (Fig. 2c; Additional file 2: Note VII). It is therefore crucial to assess the tumorigenic potential of these variants, especially when they affect genes that are—or may be—therapeutic targets. We reasoned that several features of each specific mutation as well as of the genes they affect could help address this question. Moreover, we propose that some of these features of interest can be extracted from the analyses of large sequenced cohorts of healthy and tumor tissue [4, 17]. Examples of relevant attributes include the following: i) the mode of action of the gene in the cancer type (oncogene or tumor suppressor); ii) the consequence type of the mutation (e.g., synonymous, missense, or truncating); iii) its position within the transcript; iv) whether it falls in a mutational hotspot or cluster; v) its predicted functional impact; vi) its frequency within the human population; and vii) whether it occurs in a domain of the protein that is depleted of germline variants. The CGI assesses the tumorigenic potential of the variants of unknown significance via OncodriveMUT, a newly developed rule-based approach that combines the values of these features (Fig. 2d; Additional file 2: Note IVa). We assessed the performance of OncodriveMUT in the task of classifying driver and passenger mutations, using the Catalog of Validated Oncogenic Mutations (*n* = 5314) and a collected set of likely neutral—i.e., non-tumorigenic—PAMs affecting cancer genes (*n* = 1676). We found that OncodriveMUT separated the variants of these two data sets with 86% accuracy (Matthews correlation coefficient, 0.64), out-performing other methods employed for this goal (Additional file 2: Note IVb). In addition, for several features, the variants classified as drivers by OncodriveMUT followed the trend expected for oncogenic mutations (e.g., they exhibited larger clonal fractions among all mutations in cancer genes), and OncodriveMUT’s predictions on a set of recently probed uncommon cancer mutations exhibited a high concordance with experimental evidence [18–21] (Additional file 2: Note IVb). Of note, the attributes employed by OncodriveMUT to classify each variant are detailed in the CGI output, which facilitates the user’s review of the results. In summary, the CGI annotates the mutations affecting cancer genes with features relevant to their potential role in cancer, identifying validated oncogenic events and identifying the most likely drivers among the variants of unknown significance.

### A database of genomic determinants of anti-cancer drug response

**Fig. 3:**
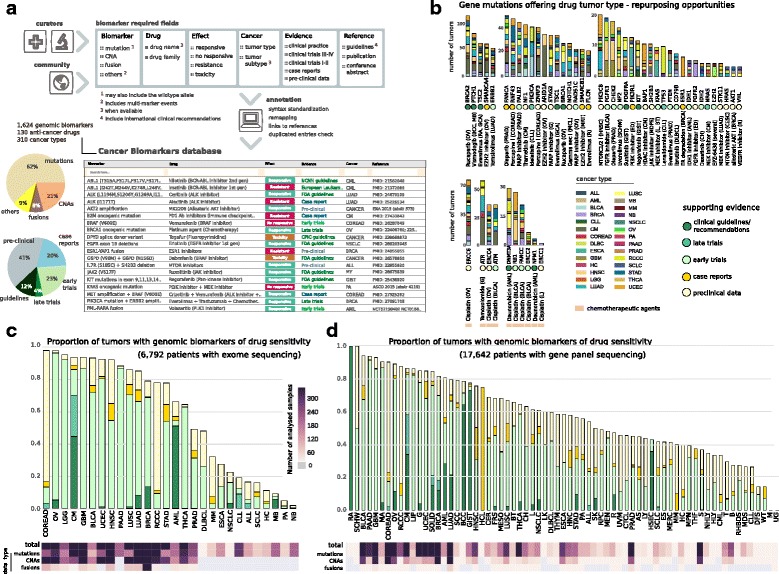
Cancer Biomarkers Database. **a** A board of clinical and research experts gather the genomic biomarkers of drug response to be included in the Cancer Biomarkers database through periodic updates. The *upper panel* displays the simplified schema of the data model. The clinical/research community is encouraged to provide feedback to edit an existing entry or add a novel one by using the comment feature available in the web service. Any suggestion is subsequently evaluated by the scientific team and incorporated as appropriate. A semi-automatic pipeline annotates any novel entry to ensure the consistency of the attributes, including variant re-mapping from protein to genomic coordinates when necessary. The *lower panel* displays some of the 1574 biomarkers that have been collected in the current version of the database, and the pie charts summarize the content. *CNA* copy number alteration. **b** CGI analyses detect putative driver mutations in individual tumors that are rarely observed in the corresponding cancer type. When these variants are known targets of anti-cancer therapies, they may constitute tumor type re-purposing opportunities. The graph summarizes some of these potential opportunities detected by the CGI on 6792 pan-cancer tumors with exome-sequencing data, which are currently unexplored. The barplots display the overall number of tumor samples (separated by cancer type) in which they were observed. The acronym of the cancer type in which the genomic event is demonstrated to confer sensitivity to the drug is shown in parentheses following the name of the drug, and the clinical evidence of that association is represented through *color circles* (note that the clinical guidelines/recommendations label refers to FDA-approved or international guidelines). Targeted drugs and chemotherapies are shown separately. Cancer acronyms that are not included in the Fig. 2 legend: *RA* renal angiomyolipoma, *BCC* basal cell carcinoma, *GCA* giant cell astrocytoma, *G* glioma, *MCL* mantle cell lymphoma, *MRT* malignant rhabdoid tumor, *R* renal, *CH* chollangiocarcinoma. **c** Therapeutic landscape of 6792 tumors with exome-sequencing data. Fraction of tumors with genomic alterations that are biomarkers of drug response in each cancer type. Colors in the bars denote the clinical evidence supporting the effect of biomarkers in that disease (see evidence colors in b). Note that the event with evidence closest to the clinical evidence is given priority when several biomarkers of drug response co-occur in the same tumor sample. The lower part of the graph indicates the total number of samples per cancer type, detailing the number of samples in which mutation, CNA, and/or fusion data were analyzed. Cancer acronyms as in the Fig. 2 caption. **d** Same as c for a cohort of 17,462 tumors sequenced by targeted panels and gathered by the GENIE project. Tumors were grouped according to the most specific disease subtype available in the patient information. Cancer acronyms that are not included in the Fig. 2 legend are detailed in Additional file 2: Supplementary content

The second major aim of the effort to interpret cancer genomes is to identify which tumor alterations may shape the response to anti-cancer therapies. Knowledge on the influence of genomic alterations on drug response is continuously generated and reported through publications, clinical trials, and conference communications. Nevertheless, collecting and curating relevant information into an easy-to-use resource supporting the comparison with newly sequenced tumors and organize the results according to the needs of different users is challenging. The CGI employs two resources to explore the associations between gene alterations and drug response. The first is the Cancer Biomarkers database, an extension of a previous collection of genomic biomarkers of anti-cancer drug response [12] which currently contains information on 1624 genomic biomarkers of response (sensitivity, resistance, or toxicity) to 310 drugs across 130 types of cancer. Negative results of clinical trials, e.g., the unsuccessful use of BRAF V600 inhibitors as a single therapeutic agent in colorectal cancers bearing that mutation, are also included in the database. Importantly, these biomarkers are organized according to the level of clinical evidence supporting each one, ranging from results of pre-clinical data, case reports, and clinical trials in early (I/II) and late phases (III/IV) to standard-of-care guidelines. The database is continuously updated by a board of medical oncologists and cancer genomics experts (Fig. 3a; Additional file 2: Note V). As explained in the “Introduction”, the Cancer Biomarkers database is only one of the resources currently annotating the biomarkers of tumor response to drugs (Additional file 1: Table S1). The leading institutions developing these knowledgebases were recently integrated into the Variant Interpretation for Cancer Consortium (http://cancervariants.org/) under the umbrella of the Global Alliance for Genomics & Health [22]. Besides the aggregation of the data collected by each individual resource, the aim of this project will be to establish community standards to represent and share this information.

The second resource is the Cancer Bioactivities database, which currently contains information on 20,243 chemical compound–protein product interactions that may support novel research applications. We built this database by compiling a catalog of available results from bioactivity assays of small molecules interacting with cancer proteins. This information was obtained by querying several external databases (Additional file 2: Note VI). The CGI matches the alterations observed in newly sequenced tumors to the biomarkers or target genes in these two databases. This process supports the identification of biomarkers at different levels of gene resolution, ranged from variants affecting a gene region to specific amino acid changes. Of note, the CGI also reports co-occurring alterations that affect the response to a given treatment as appropriate. This includes the co-existence of biomarkers of resistance and sensitivity to the same drug, and biomarkers of drug sensitivity that depend upon simultaneous genomic events. In summary, these two databases constitute comprehensive repositories of genome-guided therapeutic actionability in cancer according to current supporting evidence. Both resources are available for download through the CGI website (https://www.cancergenomeinterpreter.org/biomarkers, https://www.cancergenomeinterpreter.org/bioactivities).

## Utility and discussion

The CGI (and the databases created to support its implementation) are distributed under an open license, and the resource can be accessed via its web site at https://www.cancergenomeinterpreter.org and through an Application Programming Interface (API; Additional file 2: Notes Ic and Id). The use of the CGI to automatically interpret cancer genomes has broad potential applications, ranging from basic cancer genomics to the translational research setting. One feature of the CGI that makes it particularly suitable for different types of applications is its usability. The user can input the tumor alterations to be analyzed by uploading files following different standards and/or by typing them in a free-text box. The system is prepared to automatically recognize and re-map as necessary [23] different formats, such as genomic, transcript, or protein-based coordinates for mutations [23] (Additional file 2: Note Ib). The use of the CGI can help addressing questions raised in different oncology research settings. A newly sequenced group of tumors may be readily interpreted, and systematic analyses of large sample sets are supported as exemplified with the 6729 pan-cancer cohort presented in this article. The application of the CGI to the mutations profiled across the whole exomes of these tumors delivered a catalog of putative driver alterations across its 28 cancer types (made available through http://www.intogen.org; Additional file 2: Note VII). The potential of a comprehensive analysis of individual alterations is illustrated by the identification of uncommon events in a tumor cohort that may be exploited by drug re-purposing opportunities (Fig. 3b; Additional file 2: Note VII). Overall, the CGI identified 5.2 and 3.5% of the tumors with genomic alterations that are biomarkers of drug response used in the clinical practice (FDA-approved or international guidelines) or reported in late phase (III–IV) clinical trials, respectively. When considering biomarkers supported by lower levels of clinical relevance, a total of 62% of the tumors exhibited at least one biomarker with increased response to an anti-cancer drug according to findings in early clinical trials, case reports, or pre-clinical assays. These numbers varied greatly across tumor types, partially explained by the relevance of cancer-recurrent alterations in shaping the response to drugs, such as inhibitors of the BRAF V600 mutated form in cutaneous melanoma (clinical guidelines), certain chemotherapies administered for DNMT3A or NPM1 mutant acute myeloid leukemias (clinical guidelines), PIK3CA mutation inhibitors in breast cancer (early clinical trial results [24]), and WEE1 inhibitors in TP53 mutated ovary tumors (early clinical trial results [25]) (Fig. 3c; Additional file 2: Note VII). However, this cohort mostly includes samples sequenced at diagnosis and thus they may not reflect the type of tumors that are evaluated by molecular oncology boards at present. We therefore also applied the CGI to the sequencing data of 17,642 tumors recently released by the GENIE project, which profiled more clinically advanced cancers using targeted panels [26]. The CGI identified a larger percentage of tumors bearing potential actionable genomic alterations in that cohort. Specifically, 8 and 6% of GENIE tumors exhibited biomarkers of drug response used in clinical practice or reported in late clinical trials, and overall 72% of these tumors exhibited at least one alteration reported as a biomarker of drug response supported by any level of clinical evidence (including pre-clinical data; Fig. 3d; Additional file 2: Note VII). These percentages do not include cases in which a tumor exhibits co-occurring alterations that confer resistance to a given drug, in which the therapy was not *in silico* prescribed accordingly. Of note, the GENIE cohort exhibited a larger number of genomic biomarkers of drug resistance (to both targeted therapies and immune checkpoint blockade agents), as expected of tumors with a higher proportion of recurrence/relapse patients (Additional file 2: Note VII). These analyses provide a comprehensive state-of-the-art snapshot of the putative genomic drivers of cancer and the landscape of genomic-guided therapies according to our current knowledge. In addition, the application of the CGI to analyze the results of drug responses observed in tumors with different genomic architecture can facilitate the discovery of novel genomic biomarkers of drug sensitivity or resistance. The distinction between driver and passenger events recently contributed to the development of better predictive models to identify novel genomic markers of drug response in cancer cell lines [27].

In previous examples, the systematic analysis of large datasets was facilitated by the automatic classification of cancer variants that CGI provides. However, the detailed review of these results is empowered by the inclusion in the output reports of all the annotations employed by the CGI. The ability to review these data is especially critical in the clinical research setting. In this case, the use of the CGI to analyze the list of alterations detected in a patient’s tumor could support decision-making in multiple scenarios, assessing variants of unknown significance that may have implications for response to therapy. Early clinical adopters of the CGI have used the resource to support final decisions about the most appropriate genomic-guided clinical trial to enroll cancer patients or explore potential drug re-purposing opportunities for pediatric tumors unresponsive to standard-of-care therapy (see these use cases in Additional file 2: Note VIII).

Crucial to the performance of the CGI are the maintenance and further development of its two distinct types of resources: the repositories of accumulated knowledge, which are continuously generated, and the bioinformatics methods to estimate the relevance of those events that are not yet validated. As new tumor cohorts are re-sequenced and analyzed, more comprehensive catalogs of cancer genes and oncogenic mutations will be obtained, including both new malignancies and new genomic elements. In particular, the possibility to identify non-coding cancer drivers [28] from currently generated whole-genome sequencing data will open up the opportunity to explore the actionability of non-coding genomic alterations (https://dcc.icgc.org/pcawg). With respect to the aggregation, curation, and interpretation of the relevance of cancer variants, our team follows the standard operating procedures developed under the umbrella of the H2020 MedBioinformatics (http://www.medbioinformatics.eu/) project, thus ensuring the mid-term maintenance of these resources. Feedback from the community is also facilitated through the CGI web interface. Access to this type of cancer data is crucial for the advance of precision medicine, but is highly complex for a single institution to comprehensively manage and update. We envision that individual databases will continue to be maintained to fulfill specific needs [11], but our long-term impact will largely rely, first, on the establishment of international standards for the collection of data relevant to associations between cancer variants and clinical outcomes and, second, on our collective success in encouraging the community to share and harmonize such knowledge.

## Conclusions

The CGI is a versatile platform that automates the steps proposed here for the interpretation of cancer genomes. It annotates the alterations detected in human tumors with features that may inform about their involvement in tumorigenesis. It also highlights the alterations of the tumor that constitute biomarkers of response to anti-cancer drugs, according to current levels of evidence. The CGI is easy to use, and will improve with new knowledge extracted from the study of thousands of tumors. We envision that it will become established as a useful tool in both the basic and translational cancer research settings.

## Additional files

Additional file 1: **Table S1.** The features provided by different resources/methods and details about which of them are employed by the CGI. (XLS 21 kb)

Additional file 2: Supplementary methods, use cases description, **Table S2.** and **Figures S1–S3.** (PDF 6267 kb)
