# Supplementary material for: Cancer Genome Interpreter annotates the biological and clinical relevance of tumor alterations
Source: Genome Med. 2018 Mar 28;10:25. doi: 10.1186/s13073-018-0531-8 (PMC5875005; doi:10.1186/s13073-018-0531-8)
Supplement: Supplementary file 2 — Supplementary methods, use cases description, Table S2. and Figures S1–S3. (PDF 6267 kb) [file 13073_2018_531_MOESM2_ESM.pdf]

Cancer Genome Interpreter annotates the biological and clinical relevance of tumor alterations

Supplementary Material

Supplementary Material

Table of Contents

I. Cancer Genome Interpreter platform....3
Ia. Overview....3
Ib. Pipeline annotations....4
Ic. Web interface....5
Id. Application Programming Interface....5
II. Catalog of Cancer Genes....6
III. Catalog of Validated Oncogenic Mutations....8
IV. OncodriveMUT....9
IVa. Overview....9
IVb. Benchmarking....10
V. Cancer Biomarkers Database....12
VI. Cancer Bioactivities Database....13
VII. Use of the CGI in pan-cancer sequenced cohorts....14
VIII. The CGI in the support of clinical decision-making....16
References....17
Supplementary Table 2....19
Supplementary Figures....21
Figure Legends....24
Supplementary legend for Figure 3....26

I. Cancer Genome Interpreter platform

Ia. Overview

The Cancer Genome Interpreter (CGI) first identifies the genomic alterations (mutations –i.e. point substitutions or small insertions/deletions–, copy number alterations and/or gene translocations) driving the tumor growth. On detail, each mutation is classified as (i) a known oncogenic mutation in the tumor; (ii) a known oncogenic mutation in other cancers; (iii) a predicted driver mutation of the tumor (these are further divided into two tiers); (iv) a predicted passenger event; (v) a variant which does not affect the protein sequence or (vi) a polymorphism (i.e. major allele frequency greater than 1% across healthy donors[1]). Each gene amplification or deletion is classified as (i) a known oncogenic copy number alteration (CNA) of the tumor; (ii) a known oncogenic CNA in other cancers; (iii) a predicted driver CNA of the tumor; or (iv) a predicted passenger event. Finally, each translocation is classified as (i) a known oncogenic event of the tumor; (ii) a known oncogenic event in other cancers; or (iii) a translocation of uncertain significance. These analyses are supported by an ensemble of databases and bioinformatics methods based on several existing or newly developed resources (see the Catalog of Cancer Genes, the Catalog of Validated Oncogenic Mutations and the OncodriveMUT method sections in the present document for further details). Of note, the system assumes that all genomic alterations are correctly called (e.g. genes with unclear copy number status boundaries or mutations with low quality calls) and entered by the user.

Thereafter, the CGI explores potential therapeutic opportunities offered by the tumor's genomic makeup. Tumor alterations are compared with genomic biomarkers of anti-cancer drugs response (sensitivity, resistance and toxicity) annotated in the Cancer Biomarkers database (see section V for further details). The CGI matches this information to the alterations observed in the tumor taking into account several considerations. First, it detects and groups co-occurring alterations that are known to interact in the response to a given drug. This includes the co-occurrence in the tumor of biomarkers of resistance and sensitivity to the same drug. Second, the match between the observed genomic alteration and the– biomarker of drug response takes into account the level of detail on the latter, e.g. –in the case of mutations– the system distinguishes whether the biomarker refers to any mutation in the gene, in one particular exon (or domain) or a specific aminoacid change . The results of the alteration analysis step are considered here; e.g the OncodriveMUT classification of a variant is taken into account for the *in silico* prescription in the case of biomarkers that are solely defined as an oncogenic mutation of a given gene. ; Finally, the *in silico* prescription considers possibilities of two types of repurposing of anti-cancer drugs. The cancer-type repurposing is used for cases in which the alteration observed in the tumor has been described as a biomarker of response to the drug in a tumor type that is different to that of the sample(s) under analysis, following the hierarchy of tumor types taxonomy. The alteration-type repurposing describescases in which a different

alteration than the one described in the biomarker, but with the same putative effect, is observed in the tumor (e.g. a deletion of a tumor-suppressor when the biomarker is a loss-of-function mutation).

Furthermore, the CGI also explores as potentially interesting compounds that have been shown experimentally to bind to the products of genes with driver alterations in the tumor sample. This is based on the information of the Cancer Bioactivities database, which collects data of gene-compound chemical interactions (see the section VI for further details). This process takes into account (i) the experimentally measured strength of the reported interaction; and (ii) whether the mechanism of action of the compound on the targeted gene is coherent with the mode of action of the latter (i.e. inhibitors for oncogenes, and agonists for tumor suppressors).

All CGI analyses are cancer-specific and thus the tumor type of the sample(s) to analyze is required as an input. The CGI uses an in-house cancer taxonomy which takes into account the disease hierarchy (e.g. mutations that are known to be oncogenic in non-small cell lung carcinomas will produce a 'tumor type match' when observed in a lung adenocarcinoma sample). Therefore, the more generic is the tumor type supplied for the sample to be analyzed, the less specific the results of the CGI will be.

Ib. Pipeline annotations

The input of the CGI consists in a list of genomic alterations detected in one (or more) tumor sample(s). The CGI is able to analyze mutations (point substitutions and small insertions/deletions), gene CNAs and/or translocations. The system accepts and automatically recognizes several formats, including Human Genome Variation Society (HGV, either in genomic or protein coordinates) and Variant Call Format (VCF) for mutations. Direct or inverse mapping between genomic and protein coordinates of mutations is supported by the TransVar method[2]. To annotate the mutations, the CGI selects the transcript with the longest CCDS sequence (or longest cDNA sequence if multiple CCDS transcripts of the same length exist or the gene has no CCDS transcript), according to data retrieved from Ensembl v70, except for a set of 109 genes the canonical transcript of which was manually selected. The CGI reports include several mapping attributes such as the exon and the Pfam[3] domain affected by the mutated residue. Importantly, data provided by different databases included in the CGI (e.g. the aggregated data to build the Catalog of Oncogenic Variants) is consistently re-annotated using identical syntax and versions in order to guarantee internal compatibility. Therefore, the CGI pipeline re-maps the mutations introduced by the user accordingly to guarantee appropriate cross-matches.

Ic. Web interface

The CGI framework is freely available on the web at http://cancergenomeinterpreter.com. As stated before, the input of the CGI is (a) the genomic alterations of the tumor/s; and (b) its cancer type. The latter can be selected from a taxonomy tree that follows the in-house cancer classification. Note that several tumor samples can be analyzed in a single CGI run as far as they belong to the same tumor type, since the analysis is cancer-specific. The list of alterations may be provided as (i) one (or more) tab-separated files; and/or (ii) via a free text box. Once the user executes a new analysis, the process may be tracked using the identifier assigned to it by the system, and --once completed-- the results are stored during 48 hours. The execution time of each analysis depends on (i) how long the job takes to get a slot in our computer cluster, (ii) the time required to load the data structures used by the CGI, and (iii) the number of entries to analyzed. With the aim of reducing the overall time in some of these analyses, the results for the most frequent alterations observed in tumors are pre-computed. Once finished, the resulting CGI output is provided via a web report that can be interactively browsed and filtered. This report is divided into two parts. The first one presents the result of the alterations analysis (which may be further divided into three tabs containing the results of mutations, CNAs and/or translocations as appropriate) and the other with the *in silico* prescription (organized in a tab showing the match of the tumor with the Cancer Biomarkers database and another tab showing the match with the Cancer Bioactivities database). If the user logs into the system, these reports are stored in a *Results* page within the CGI website associated with that user's account. The login process only requires a valid email address and the access is thereafter immediately granted. The CGI reports may be shared by creating a unique link and the results may be downloaded as tab-separated files. To prevent unauthorized access or disclosure, to maintain data accuracy, and to ensure the appropriate use of information, CGI uses a range of reasonable physical, technical, and administrative measures to safeguard the information, in accordance with current technological and industry standards. In particular, all connections to and from our website are encrypted using Secure Socket Layer (SSL) technology. The CGI never has access to users' password and uses a trusted third party protocol to authenticate the user. While the analyses are running, they are stored in our private servers. The results can be downloaded, shared or deleted and they are organized by an editable title. When a CGI analysis is deleted, it is completely and permanently removed from the servers.

Id. Application Programming Interface

The CGI resource can also be accessed programmatically by an API created via REST. Only registered users can make use of the API, since a token is needed for any communication between the end user and the REST API. Further details can be found at https://www.cancergenomeinterpreter.org/rest_api

II. Catalog of Cancer Genes

The CGI focuses the analysis on genomic alterations that affect the genes thought to be potentially involved in the pertinent cancer type. Although all the variants are annotated with relevant attributes (see section IV), only those affecting cancer genes qualify for further consideration as potential driver events. The Catalog of Cancer Genes is a collection of genes driving tumorigenesis in a certain tumor type(s) upon a certain alteration type (mutation, CNA and/or gene translocation). This information is supported by (a) validated data; and/or (b) bioinformatics prediction. For the former, known cancer genes are collected from the following manually curated resources: (i) the Cancer Gene Census[4]; (ii) genes bearing mutations known to lead to tumor phenotypes (see the Catalog of Validated Oncogenic Mutations); and (iii) genes with validated alterations that confer increased sensitivity to targeted anti-cancer drugs (see the Cancer Biomarkers database). The cancer type names used in the original sources are translated as appropriate to the in-house CGI tumor taxonomy.

The Catalog of Cancer Genes also contains putative driver genes identified by bioinformatics analyses of large tumor cohorts resequenced by consortia such as The Cancer Genome Atlas, and the International Cancer Genome Consortium. The identification of mutational driver genes was carried out through the combination of three orthogonal signals of positive selection across each tumor cohort, namely, the frequency of the mutations, a bias of mutations towards high functional impact and their spatial clustering along the protein sequence[5,6]. For the identification of CNA driver genes, we first collected genes located within chromosomal regions that suffer recurrent focal amplifications or deletions across the samples of each tumor type[7]. Second, the CNAs were required to be coherent with the (predominant) mode of action of the gene, i.e. deletions for known (or predicted, see below[8]) tumor suppressors and amplifications for known (or predicted) oncogenes. Note that both alteration types are accepted if the gene has an ambiguous/uncertain role (see below) for that cancer type. Finally, only genes with a significant change in expression coherent with the copy number change (up-regulation for gene amplifications, down-regulation for gene deletions) were finally nominated as the predicted drivers upon CNAs of that cancer type. The misregulation was evaluated via the comparison of RNAseq values of the group of samples diploid for the gene locus *versus* samples with the CNAs (only homozygous deletions or multi-copy gains were included).

At present, these analyses have been carried out across a 6,792-overall samples pan-cancer cohort comprising 28 different tumor types.

Finally, the mode of action (loss-of-function *versus* gain-of-function) of each cancer gene has been also included in the Cancer Genes Catalog. This information can be (a) validated and as such, obtained from manually curated resources; or (b) predicted via bioinformatics analyses[8]. Of note, the mode of action includes an 'ambiguous' role, which is stated when it is not known and it can not be predicted with reliability

by the computational methods employed to estimate so or the gene acts as both a tumor suppressor and an oncogene in a context-dependent manner.

III. Catalog of Validated Oncogenic Mutations

Not all mutations identified in cancer genes are capable of driving tumorigenesis. Consequently, the CGI considers whether a gene is mutated, but also which particular variant occurs. Therefore, we first compiled an inventory of mutations in cancer genes that are demonstrated to drive tumor growth or predispose to cancer. This was retrieved by combining the data contained in the DoCM[9] , ClinVar[10] and OncoKB[11] databases as well as the results of several published experimental assays, as those compiled by Martelotto et al.[12]. We also considered as oncogenic the mutations reported to increase sensitivity to targeted drugs included in the Cancer Biomarkers Database (see below). Germline variants found to predispose to cancer, which we retrieved from the ClinVar and IARC resources[10,13], were also included. Contradictory data (i.e. a variant stated as oncogenic and neutral by different resources) was flagged and filtered out. In all, 24 variants (0.4% of the total) were filtered out due to this. The current version of the Catalog of Validated Oncogenic Mutations includes 5,610 somatic/germline oncogenic variants. This dataset is available at https://www.cancergenomeinterpreter.org/mutations. When this information was matched to the somatic mutations identified by exome-sequencing in the 6,792 samples pan-cancer cohort (see main text of the manuscript), we found that only a minority of the mutations observed across cancer genes were validated oncogenic events. Thus, a majority of the protein-affecting mutations (~88%) observed in tumors, even if they occur in well known cancer genes, are of unknown significance, highlighting the need for tools to classify them (see the OncodriveMUT section). Of note, we observed some of these validated oncogenic events in cancer types in which they had not been described before, such as DNMT3A p.R882H, SF3B1 p.K700R and JAK2 p.V617F mutations (known in blood malignancies[14–16]) in breast, renal and glioblastoma tumors in the pan-cancer cohort, respectively. These rare events may be further relevant when they are involved in the response to anti-cancer drugs.

IV. OncodriveMUT

IVa. Overview

We have developed a novel method, OncodriveMUT, with the aim of gaining further insights into the oncogenic potential of the mutations of unknown significance. OncodriveMUT is used by the CGI to analyze the mutations in cancer genes that are not found in the Catalog of Validated Oncogenic Mutations. OncodriveMUT combines measurements performed at the level of each individual mutation with knowledge about the driver genes (or regions thereof) in which these mutations are found. This knowledge is retrieved from the analysis of large cohorts of sequenced tumors and healthy donors, which provides the statistical power to discover gene features that are relevant to assess the importance of particular mutations. At present, we have analyzed cohorts of tumors (6,792 samples across 28 cancer types[5]) and samples from healthy donors (60,706 unrelated individuals)[17]. On detail, the knowledge retrieved from cohorts of healthy donors are the allele frequency of variants and the protein domains depleted of functional variants in the general population. The latter points out to protein regions that may be less tolerant to functional variants. To identify them, we searched for protein domains (from the Pfam[3] database) enriched by very rare (1 out of 10,000 samples) variants according to ExAC data[17]. As a result, we identified 94 genes exhibiting 24 types of so-called '*delicate domains*', which include the tyrosine phosphorilation, the protein kinase, the homeobox and the SH2 domains. On the other hand, the analysis of sequenced tumor cohorts yielded: (i) the signals of positive selection of each gene in each tumor type[5], which is the cornerstone to identify cancer genes; (ii) the mode of action of each cancer gene in tumorigenesis, i.e. loss-of-function, oncogene or ambiguous[8]; and (iii) protein sites with an unexpectedly high concentration of somatic mutations, i.e. mutation clusters[18]. Finally, as mutation-centric features, the OncodriveMUT uses (i) their consequence type, i.e. missense , inframe indel, or truncating mutation (e.g. a mutation within a canonical splice site, a frameshift variant or the insertion of a premature stop codon); the location of the mutation in terms (ii) of the domain (to match it to the list of delicate domains, see above), and (iii) of the protein site, on detail whether it occurs before the last exon-intron junction (which is more likely to trigger the nonsense-mediated decay pathway in case of a truncated protein) or in the last portion of the protein (since disrupting mutations may be less deleterious if they occur at the very last protein sites); and (iv) the estimated deleteriousness of the mutation, measured by the Combined Annotation Dependent Depletion score[19].

OncodriveMUT combines these measurements using a set of heuristic rules, which are shown in Supplementary Table 2. We compared the performance obtained by these rules with a machine-learning approach; to do so, we built a random forest machine learning and classification algorithm[20]. Using *bona fide* oncogenic mutations and neutral events observed across cancer genes (see below), a random-forest classifier with 1,000 estimators was trained in a ten fold cross-validation with 70% of the features in order to predict the remaining 30% (data not shown). Both, the machine-learning and the heuristic approach exhibited similar

performance. We therefore decided to use the latter, since the rationale behind the classification of each variant by OncodriveMUT is then human-readable and the critical review of these results is facilitated. To empower the user to carry out such a review, the measurements and attributes of each variant employed by the OncodriveMUTclassification are included in the CGI output reports. In addition, these data can support further exploration of the mode of action of each mutation. For instance, most inframe indels detected as driver events in tumor suppressor genes (whose effect is more difficult to estimate than their clearly more deleterious frameshift relatives) occur within regions where somatic mutations tend to cluster in these genes. This may suggest a loss-of-function mechanism driven by the disturbance of critical protein sites (e.g. inframe deletions in CDKN2A binding sites within the second exon[21]), or the acquisition of dominant-negative phenotypes driven by the creation of particular protein fragments (e.g. inframe indels in the 5th exon of TP53[22]). The incorporation of additional computational measurements developed in the future, as well as the study of novel data and experimental results, will help to further improve OncodriveMUT analyses.

IVb. Benchmarking

First, *bona fide* driver and passenger mutations in cancer genes were collected to be used as positive and negative data sets to benchmark the OncodriveMUT approach, respectively. The former was composed of the entries gathered in the Catalog of Validated Oncogenic Mutations (n=5,314). For the latter, we collected a set of protein affecting mutations observed in cancer genes and found to be non-pathogenic and/or neutral in terms of oncogenesis (according to ClinVar and OncoKB annotations[10,11], n=670) or common polymorphisms (major allele frequency larger than 1% in the general population according to ExAC[17], n=1,006). As a result, we found that OncodriveMUT separates the variants of these two data sets with 86% of accuracy (Matthews correlation coefficient, 0.64) (Suppl. Figure 1A). OncodriveMUT outperformed other methods developed with similar purpose[19,23–26] (Suppl Figure 2). In addition, several data sets were collected to assess whether the mutations classified as drivers by OncodriveMUT follow *a priori* expected behaviors of oncogenic mutations. First, we downloaded the frequency of somatic protein affecting mutations in cancer genes observed across tumor samples from COSMIC v76[27]. Second, the major allele frequency across the general population of germline variants leading to a change of protein sequence in cancer genes was retrieved from ExAC[17]. And third, the cancer cell fraction of mutations observed in cancer genes was calculated using their variant allele frequency corrected by the estimated tumor purity and gene copy number[5]. As a result, we observed that mutations classified as drivers by OncodriveMUT are enriched amongst recurrent COSMIC mutations (Suppl. Fig. 1B). They are also enriched for rare germline variants across healthy donors (Suppl. Fig. 1C). Both results are expected from oncogenic events. However, a certain degree of circularity in this validation must be noted, as one of the features used by OncodriveMUT is whether the mutation under analysis falls within a cluster of somatic mutations previously identified using available sequenced tumor cohorts. On the other hand, mutations in cancer genes classified as drivers by OncodriveMUT exhibit larger cancer cell fraction than those classified as passengers (Suppl. Fig. 1D), as

expected from events that undergo positive selection within the cancer cell clonal population. Of note, only protein-affecting mutations in cancer genes were considered in these tests, which highlights the ability of the OncodriveMUT method to point out those with more oncogenic potential.

Finally, we gathered results from several available experimental assays evaluating the effect of cancer mutations to assess the agreement of OncodriveMUT with the experiment in completely independent test sets. First, we used all possible missense mutations along the protein sequence of TP53 and their functional effect evaluated in yeast assays[28]. On detail, this study measured the transactivation of the TP53 mutants on several reporter genes. Only activities lower than 140% (activity of the mutant in relation to the wild-type) were included. Second, the effect of rare mutations (i.e. lowly recurrent across cancer patients) in several oncogenes were collected from three recent studies. We considered oncogenic (i) PIK3CA-mutants exhibiting activity in all the six experiments provided in ref. [29] -regardless of their strength–; (ii) mutations in oncogenes leading to sustained tumor growth before 130 days in the *in vivo* experiments provided in ref. [30]; and (iii) mutations in oncogenes validated as tumorigenic in the functional screens performed in ref. [31]. Of note, any mutation included in the positive or negative sets described in the first paragraph was filtered out from this step to avoid redundancy between the two evaluations. As a result, (a) TP53 mutants classified by OncodriveMUT as driver mutations exhibited larger impairment of the gene activity than those predicted as passengers (Suppl. Figure 1E); and (b) OncodriveMUT classification of rare mutations in cancer genes reached an 82% of agreement with the experiments (Suppl. Figure 1F).

V. Cancer Biomarkers Database

The Cancer Biomarkers Database is a manually curated resource collecting genomic biomarkers of drug response found in cancer patients or in pre-clinical assays. This database follows the organization proposed in the Gene Drug Knowledge Database (GDKD)[32], which requires, among others, the evidence supporting each alteration-drug association. On detail, five distinct levels of supporting evidence are employed: (a) clinical guidelines, which includes FDA-approved indications and recommendations from international organizations such as NCCN; (b) late clinical trials (i.e. phases III-IV); (c) early clinical trials (i.e. phases I-II); (d) clinical case reports; and (e) pre-clinical data. Genomic alterations in the database may be biomarkers of increased sensitivity, resistance or toxicity to anti-cancer therapies. Of note, negative evidences, i.e. those alterations that do not affect the response to a given drug (e.g. the use of BRAF V600 inhibitors as single agent in colorectal cancers bearing that mutation), were also included in the database and labeled as 'non-responsive'. Absence of an event (e.g. a wild-type allele) and multi-marker entries (e.g. PIK3CA oncogenic mutation + ERBB2 amplification for Everolimus + Trastuzumab + Chemotherapy treatment in breast cancer) are also contemplated. Each entry also includes the cancer type(s) in which this association has been demonstrated and the reference (e.g. PubMed identifier or conference abstract reference) of that study. The data is collected by a board of clinical oncologists and research experts organized by cancer type expertise, who are in charge of filling the minimum-required fields for each new entry following the data model. Biomarkers supported by lower-level clinical evidences (i.e. retrieved from pre-clinical assays), which are much more abundant in the literature, are selected based on the robustness of the supporting data and their potential to be translated into a clinical trial. Thereafter, each new biomarker entry is annotated using a semi-automatic bioinformatics pipeline, which ensures –among other things-- the use of a systematic nomenclature and a standardized cancer taxonomy, the accuracy of the nucleic acid – amino acid system annotation equivalence, and the avoidance of duplications or inconsistent information for a given genomic alteration. The data model of the information, and the creation and maintenance of the database is currently developed under the umbrella of the H2020 MedBioinformatics project (http://www.medbioinformatics.eu/). The Cancer Biomarkers Database has been made available at https://www.cancergenomeinterpreter.org/biomarkers, which allows interactive browsing and the feedback of the community. The Cancer Biomarkers Database is currently being integrated with other resources developed with similar purpose by the Variant Interpretation Cancer Consortium (http://cancervariants.org/) under the umbrella of the Global Alliance for Genomics & Health. Besides the aggregation of the data collected by each individual initiative, this project will support the establishment of community standards to collect, organize and share this information.

VI. Cancer Bioactivities Database

The Cancer Bioactivities Database was built from ChEMBL v21[33] data on compound assays. Ensembl v70 gene symbols were mapped to uniprot IDs, through Biomart Ensembl API, and mapped to ChEMBL target IDs through the mapping file provided in ChEMBL v21 downloaded from its *ftp* server. Only genes with a valid HGNC symbol were considered. Next, we retrieved all bioactivity data associated to the target-molecule interactions reported by all assays probing the interaction. We included assays that measured a *confidence score* higher than or equal to 4 when this information was available, and entries suggesting errors in the annotations (*data validity comment* field) were filtered out. We considered bioactivities concerning the affinity of binding, the effective concentration, the efficacy of inhibition and the efficacy of competitive antagonism (IC50, EC50, Ki, Kd and Kb), whose values were converted to pActivity as appropriate. Each target-compound bioactivity was finally obtained by averaging the values across the available assays accomplishing our inclusion criteria. The resulting values were then grouped into three categories: (i) highly potent, with a binding affinity higher than 1 nM (pActivity >= 9); (ii) potent, with a binding affinity between 1µM and 1nM (9 < pActivity >= 6); and (iii) weak, with a binding affinity between 1mM and 1µM (6 < pActivity >= 3). Additional information on chemical compounds was collected, including their market status (e.g. approved or pre-clinical) and their mechanism of action (MOA). If the MOA was not available, we considered the compound as an inhibitor of the target. We grouped all MOA categories into two groups depending on whether they have a positive effect on the target (e.g *agonist* or *opener* labels) or negative (e.g *inhibitor* or *blocker* labels). The CGI *in silico* prescription includes a match column stating whether the MOA of the compound is coherent with the mechanism to drive the tumorigenesis (known or predicted) of the cancer gene, i.e. tumor suppressors for positive MOAs and oncogenes for negative MOAs.

VII. Use of the CGI in pan-cancer sequenced cohorts

We exemplify the ideas on the interpretation of cancer genomes described in this commentary through their application to a pan-cancer cohort of 6,792 exome-sequenced tumors[5]. First, we observed that 88% of the protein-affecting mutations (PAMs) observed in cancer genes (those in the Catalog of Cancer Genes) are not found in the Catalog of Validated Oncogenic Mutations and thus need further assessment to estimate their oncogenic potential. The use of OncodriveMUT to systematically address this question provides a catalog of putative driver mutations in cancer that we have made available through the IntOGen (http://www.intogen.org) resource[34]. This tool allows users to browse the driver mutations in individual tumor samples and their frequency across cancer types (Suppl. Fig. 3). Overall, the CGI analysis found that 40% of the PAMs observed in cancer genes are estimated to be passengers, with wide variation between genes. Of note, we found that the proportion of driver mutations in a tumor sample decreases as the total number of mutations increases (Pearson r=-0.15; p=1e-35). This observation is in line with the notion that the number of genomic events driving the malignancy is relatively small, even in tumors with a high mutation burden.

Second, the estimation of the oncogenic potential of the alterations are further relevant when they may provide potential targets of therapeutic intervention. The CGI *in silico* prescription showed that 62% of the tumors of this pan-cancer cohort exhibited at least one alteration reported to be a biomarker of drug response, although the majority corresponds to lower levels of clinical evidence; on detail, only 5.2% and 3.5% of the samples exhibited genomic alterations fulfilling biomarkers of drug sensitivity used in the clinical practice or reported in late (phases III-IV) clinical trials, respectively. Larger numbers of tumors carried biomarkers with lower level of supporting evidence, such as early (phases I-II) clinical trials (43%), case reports (11%) or pre-clinical data (50%). Of note, 7% of these tumors exhibited more than one genomic biomarker of drug response with a similar level of clinical relevance. This observation further stresses the importance of providing tools to prioritize their relevance, including the assessment of their clonal content when possible.

Finally, the sample-centric analysis supported by the CGI empowers the identification of alterations that are uncommon in particular tumor types but however are considered actionable in other cancers in which that alteration is observed more frequently. These events may provide potential re-purposing opportunities whose outcome is currently not known (and thus not included as a positive nor negative evidence in the Cancer Biomarkers database). Among these events, some of the most frequently observed include the possibility of targeting loss-of-function alterations of DNA damage genes and the use of rapalogs for tumors with TSC1/2 loss-of-function. Another compelling example is PTCH1, a member of the patched gene family involved in the response to hedgehog inhibitors, which are currently approved for clinical use in basal cell carcinoma and in medulloblastoma[35,36]. PTCH1 is not routinely contemplated among the genes of potential interest in other tumor types since it is rarely mutated. However, 82 samples across 19 other tumor types of the analysed pan-

cancer cohort harbored mutations estimated as drivers in this gene. Moreover, most of these tumors did not exhibit any other actionable alteration supported by strong clinical evidence. This observation may point out these PTCH1-mutated tumors as suitable candidates to be included in a potential basket trial.

Next, we compared these results with the therapeutic opportunities identified for 17,462 cancer patients profiled by the GENIE project. In comparison with the 6,792 exome-sequenced patients, the GENIE cohort is enriched for biomarkers employed by molecular oncology boards, since (a) the tumors were profiled by targeted panels designed to support the clinical programs at the participating medical centers; and (b) the project included a higher proportion of recurrent/relapsing patients and/or later stage cancers. The CGI identified 8% and 6% of tumors with biomarkers of drug response supported by clinical guidelines and late clinical trials, respectively. Biomarkers of drug response supported by data obtained in early clinical trials, case reports and pre-clinical studies were found in 49.7%, 18.7%, and 60% of patients, respectively. Overall, the CGI identified at least one biomarker of drug sensitivity supported by evidences spanning from clinical guidelines to pre-clinical data in 72% of GENIE patients, a percentage that varies across cancer types. Of note, these tumors also exhibited a considerable number of biomarkers of drug resistance, as expected from a cohort with a larger share of recurrent/relapse patients and in contrast to the 6,792 pan-cancer cohort, which is mostly composed of tumors profiled at diagnosis. Among the most recurrent events, the CGI identified EGFR T790M mutations in lung tumors (providing resistance to several EGFR inhibitors), BRAF V600E mutations in colorectal tumors (resistance to Cetuximab) and ESR1 oncogenic mutations in breast tumors (resistance to aromatase inhibitors). In addition, the CGI also identified several putative loss-of-function mutations in JAK1, JAK2 and B2M genes, which have been recently reported to confer resistance to PDL1/PD1 axis inhibitors. These mutations were found in tumors with high mutation burden and/or presenting co-occurring putative biomarkers of response to these immunotherapies (e.g. NF1 and PTEN-mutant melanomas).

In summary, the CGI provides a systematic and rapid interpretation of the genomic alterations profiled for large tumor cohorts. These analyses provide a comprehensive catalog of cancer driver variants and the *in silico* prescription refines the landscape of genomic-guided therapeutic opportunities as it stands today in newly diagnosed and advanced cancers.

VIII. The CGI in the support of clinical decision-making

The CGI has been used to support the clinical decision-making process in two clinical oncology centers that are early adopters of the resource. Vall d'Hebron Institute of Oncology is a reference medical cancer institution that routinely applies a targeted next-generation sequencing panel of –at the moment of writing this manuscript-- 60 genes designed to identify targetable mutations in the tumors of patients eligible to enroll in early clinical trials. Within this program, the systematic use of the CGI proved particularly informative in the interpretation of rare variants found in ERBB2, ERBB3, FGFR1, FGFR2, FGFR3 and FGFR4. Known oncogenic mutations in these genes are considered inclusion criteria in various clinical studies with matched targeted inhibitors that are carried out in that center; however, the decisions are not clear-cut when variants of uncertain significance are observed. Overall, out of 16 patients with ERBB2/3 mutated tumors, 5 (31%) cases (breast, colorectal and ovarian carcinomas) carried alterations previously unreported by the literature (as manually checked by the oncologists team). The CGI predicted them to be drivers, and the information provided with these results supported the final decision of the molecular tumor board to enroll the patients in clinical trials of pan-ERBB inhibitors. In the case of FGFR1/2/3/4, most of the mutations observed in the cohort were of unknown significance (7 out of 10); 5 (50%) of them (endometrial, colorectal, glioblastoma and unknown primary cancer) were predicted to be drivers by the CGI, thereby supporting the recruitment of these patients into a clinical trial for pan-FGFR inhibitors after prior revision of these results by the clinicians.

Hospital Sant Joan de Deu, a reference pediatric hospital, applied the CGI to analyze a prospective series of 18 patients diagnosed with developmental solid tumors (relapse or refractory disease in 16 of them). Most of the cases were clinically aggressive sarcomas and tumors of the central nervous system. The whole exome of the tumors was sequenced in the search for potential actionable mutations, which were subsequently confirmed by Sanger sequencing. Overall, the CGI revealed a total of 6 actionable alterations in 5 (28%) of the patients for further consideration. Of note, two of them were PTCH1 mutations predicted as loss-of-function drivers, one in a medulloblastoma and the other in a high-grade glioma, linked to the possibility of re-purposing SHH inhibitors, whose outcome was subsequently tested in a mouse model (data not shown). In summary, the CGI is a useful tool to support decision making of molecular tumor boards, such as those aimed to allocate patients to the most appropriate clinical trial or to comprehensively explore off-label opportunities for genome guided therapies in patients unresponsive to standard-of-care treatment.

Informed consent was obtained from all subjects participating in these projects, which were approved by the ethical committees of both institutions (Clinical Research Ethical committee and Research Projects commission from Hospital Universitari Vall d'Hebron and Research Projects Ethical committee from Fundació Sant Joan de Déu PIC-153-16).

References

1. Lek, M. *et al.* Analysis of protein-coding genetic variation in 60,706 humans. *Nature* **536,** 285–291 (2016).

2. Zhou, W. *et al.* TransVar: a multilevel variant annotator for precision genomics. *Nat. Methods* **12,** 1002–1003 (2015).

3. Finn, R. D. *et al.* Pfam: The protein families database. *Nucleic Acids Research* **42,** (2014).

4. Futreal, P. A. *et al.* A census of human cancer genes. *Nat. Rev. Cancer* **4,** 177–83 (2004).

5. Rubio-Perez, C. *et al.* In Silico Prescription of Anticancer Drugs to Cohorts of 28 Tumor Types Reveals Targeting Opportunities. *Cancer Cell* **27,** 382–396 (2015).

6. Tamborero, D. *et al.* Comprehensive identification of mutational cancer driver genes across 12 tumor types. *Sci. Rep.* **3,** 2650 (2013).

7. Mermel, C. H. *et al.* GISTIC2.0 facilitates sensitive and confident localization of the targets of focal somatic copy-number alteration in human cancers. *Genome Biol.* **12,** R41 (2011).

8. Schroeder, M. P., Rubio-Perez, C., Tamborero, D., Gonzalez-Perez, A. & Lopez-Bigas, N. OncodriveROLE classifies cancer driver genes in loss of function and activating mode of action. in *Bioinformatics* **30,** (2014).

9. Ainscough, B. J. *et al.* DoCM: a database of curated mutations in cancer. *Nat Meth* **13,** 806–807 (2016).

10. Landrum, M. J. *et al.* ClinVar: public archive of interpretations of clinically relevant variants. *Nucleic Acids Res.* **44,** D862-8 (2015).

11. Chakravarty, D. *et al.* OncoKB: A Precision Oncology Knowledge Base. *JCO Precis. Oncol.* 1–16 (2017). doi:10.1200/PO.17.00011

12. Martelotto, L. G. *et al.* Benchmarking mutation effect prediction algorithms using functionally validated cancer-related missense mutations. *Genome Biol.* **15,** 484 (2014).

13. Petitjean, A. *et al.* Impact of mutant p53 functional properties on TP53 mutation patterns and tumor phenotype: Lessons from recent developments in the IARC TP53 database. *Hum. Mutat.* **28,** 622–629 (2007).

14. Shih, A. H., Abdel-Wahab, O., Patel, J. P. & Levine, R. L. The role of mutations in epigenetic regulators in myeloid malignancies. *Nat Rev Cancer* **12,** 599–612 (2012).

15. Kilpivaara, O. *et al.* A germline JAK2 SNP is associated with predisposition to the development of JAK2V617F-positive myeloproliferative neoplasms. *Nat Genet* **41,** 455–459 (2009).

16. Yang, J. *et al. SF3B1 mutation is a rare event in Chinese patients with acute and chronic myeloid leukemia*. *Clinical Biochemistry* **46,** (2013).

17. Lek, M. *et al.* Analysis of protein-coding genetic variation in 60,706 humans. *bioRxiv* (2015).

18. Tamborero, D., Gonzalez-Perez, A. & Lopez-Bigas, N. OncodriveCLUST: exploiting the positional clustering of somatic mutations to identify cancer genes. *Bioinformatics* **29,** 2238–44 (2013).

19. Kircher, M. *et al.* A general framework for estimating the relative pathogenicity of human genetic variants. *Nat. Genet.* **46,** 310–315 (2014).

20. Pedregosa, F. & Varoquaux, G. *Scikit-learn: Machine learning in Python. ... of Machine Learning ...* **12,** (2011).

21. Liu, L. *et al.* Germline p16INK4A mutation and protein dysfunction in a family with inherited melanoma. *Oncogene* **11,** 405–12 (1995).

22. Brosh, R. & Rotter, V. When mutants gain new powers: news from the mutant p53 field. *Nat Rev Cancer* **9,** 701–713 (2009).

23. Wong, W. C. *et al.* CHASM and SNVBox: Toolkit for detecting biologically important single nucleotide mutations in cancer. *Bioinformatics* **27,** 2147–2148 (2011).

24. Shihab, H. a, Gough, J., Cooper, D. N., Day, I. N. M. & Gaunt, T. R. Predicting the functional consequences of cancer-associated amino acid substitutions. *Bioinformatics* **29,** 1504–10 (2013).

25. Reva, B., Antipin, Y. & Sander, C. Predicting the functional impact of protein mutations: application to cancer genomics. *Nucleic Acids Res.* **39,** e118 (2011).

26. Mao, Y. *et al.* CanDrA: Cancer-specific driver missense mutation annotation with optimized features. *PLoS One* **8,** (2013).

27. Forbes, S. *et al.* COSMIC 2005. *Br. J. Cancer* **94,** 318–322 (2006).

28. Kato, S. *et al.* Understanding the function-structure and function-mutation relationships of p53 tumor suppressor protein by high-resolution missense mutation analysis. *Proc Natl Acad Sci U S A* **100,** 8424–8429 (2003).

29. Dogruluk, T. *et al.* Identification of variant-specific functions of PIK3CA by rapid phenotyping of rare mutations. *Cancer Res.* **75,** 5341–5354 (2015).

30. Kim, E. *et al.* Systematic functional interrogation of rare cancer variants identifies oncogenic alleles. *Cancer Discov.* **2641,** 617–632 (2016).

31. Berger, A. H. *et al.* High-throughput Phenotyping of Lung Cancer Somatic Mutations. *Cancer Cell* (2015). doi:10.1016/j.ccell.2016.06.022

32. Dienstmann, R., Jang, I. S., Bot, B., Friend, S. & Guinney, J. Database of genomic biomarkers for cancer drugs and clinical targetability in solid tumors. *Cancer Discov.* **5,** 118–123 (2015).

33. Gaulton, A. *et al.* ChEMBL: A large-scale bioactivity database for drug discovery. *Nucleic Acids Res.* **40,** (2012).

34. Gonzalez-Perez, A. *et al.* IntOGen-mutations identifies cancer drivers across tumor types. *Nat. Methods* **10,** 1081–1082 (2013).

35. von Hoff, D. *et al.* Inhibition of the Hedgehog Pathway in Advanced Basal-Cell Carcinoma. *N. Engl. J. Med.* **361,** 1172 (2009).

36. Rudin, C. M. *et al.* Treatment of medulloblastoma with hedgehog pathway inhibitor GDC-0449. *N. Engl. J. Med.* **361,** 1173–1178 (2009).

Supplementary Table 2

OncodriveMUT classification details

Consequence type	Gene category	Condition	Driver prediction	Description
Missense	Tumor driver, other tumors driver	Mutation in a gene cluster	Tier 1	*Cluster - missense*
Missense	Tumor driver	CADD>25	Tier 1	*Functional - missense*
Missense	Other tumors driver	CADD>30	Tier 1	*Functional - missense*
Missense	Other tumors driver	CADD>25	Tier 2	*Functional - missense*
Missense	Tumor driver, other tumors driver	Mutation in a delicate domain and CADD>20	Tier 2	*Functional - missense*
Disrupting	Tumor driver, other tumors driver	LoF gene and the mutation is not in the distal protein portion	Tier 1	*Loss-of-function - disrupting*
Disrupting	Tumor driver, other tumors driver	Gene with ambiguous role and the mutation is not in the distal protein portion	Tier 2	*Loss-of-function - disrupting*
Disrupting	Tumor driver, other tumors driver	Mutation in a gene cluster	Tier 2	*Cluster - disrupting*
Inframe indel	Tumor driver, other tumors driver	LoF (or ambiguous) gene and CADD>25	Tier 2	*Loss-of-function - inframe*
Inframe indel	Tumor driver, other tumors driver	LoF (or ambiguous) gene and mutation in a delicate domain and CADD>20	Tier 2	*Loss-of-function - inframe*
Inframe indel	Tumor driver, other tumors driver	Mutation in a gene cluster	Tier 2	*Cluster - inframe*

This table summarizes the heuristic rules used by OncodriveMUT to classify a given variant as a potential oncogenic event. The method combines several mutation-centric features with the knowledge retrieved from the analyses of large tumor cohorts of the genes (and regions thereof) where that mutation occurs.

* the *consequence type* of the mutation; disrupting mutations include frameshift variants, insertions of a premature stop codon and mutations within canonical splice sites
* the *gene category* states whether the gene has been identified as a mutational driver of the tumor or a mutational driver of other cancers (based on experimental validations and/or bioinformatic analyses)
* the *condition* that OncodriveMUT assesses to state a mutation as driver (CADD = Combined Annotation Dependent Depletion score; LoF = validated/predicted loss-of-function mechanism of action of the gene)
* *driver prediction*: mutations classified by OncodriveMUT as drivers are divided in two different *tiers* depending on the strength of the rationale that support that statement
* the *description* column labels the OncodriveMUT classification (see below)

Each of these classifications are based in the following rationale:

Cluster missense. The majority of missense mutations observed within a mutational cluster of a gene occur in oncogene hotspots and result in a gain-of-function mechanism (e.g. BRAF V600 and KRAS G12 mutations). To a lower extent, the regional accumulation of mutations is also observed in tumor-suppressor genes, in which the mutation clusters tend to span across wider segments of the

protein sequence that may be more prone to drive a loss-of-function mechanism when targeted. An exception to the latter are those mutations that cause dominant-negative phenotypes, which tend to accumulate in specific gene sites.

Functional missense. Missense mutations outside clusters may lead to heterogeneous effects. They are prioritized on the basis of their pathogenicity score as estimated by the CADD method. Those variants exhibiting high CADD scores may lead to either gain-of-function (e.g. KRAS A59G) or loss-of-function (e.g. PTEN R159S) phenotypes. The Phred (scaled) CADD score used by OncodriveMUT as a cutoff to classify the mutations as oncogenic depends on i) whether the gene is a driver of the cancer type of the tumor under analysis or of another cancer type (less stringent criteria in the former); and ii) whether the mutation occurs within a protein domain that has been detected as *delicate* (i.e. depleted for variants in general population; less stringent criteria if this happens; note that this information is not included in the CADD method). Of note, the CADD score thresholds are selected according to their ranking within the distribution of CADD scores obtained for the whole set of possible missense mutations in the genome. Predicted driver mutations are deemed to be of tier 1 or 2 depending on the combination of these factors (see details in the table).

Loss-of-function disrupting. Disrupting mutations are likely to cause the loss of function of tumor suppressors. This may be less clear in the case of premature stop codons that are inserted after the last exon of the gene, which are less likely to trigger nonsense mediated decay mechanisms, or more in general for disrupting mutations that happen in the latest positions of the transcript (e.g. premature stop codons that cause to skip the transcription of a small gene region, or frameshift mutations that lead to incorrectly transcript the latest protein aminoacids plus the addition of a varying number of aberrant aminoacids). Therefore, the consideration of the mutation position regarding the transcript is also considered for this classification. Of note, some genes that exhibit recurrent mutations in latest transcript positions are covered by their detection as clusters (see next). Whether the gene is known to (predominantly) act as tumor suppressor in cancer or its role is ambiguous (i.e. the gene act as tumor-suppressor in a context-dependent manner or its mechanism of action cannot be unambiguously defined) leads to OncodriveMUT to classify the mutation as *tier 1* or *tier 2*.

Cluster disrupting. Disrupting mutations may also occur within regions that tend to accumulate variants. They can be clusters enriched by missense mutations or clusters that also exhibit accumulation of disrupting mutations. In the case of oncogenes, a disrupting mutation in a cluster may point out a gain-of-function driven by a particular disruption of a negative-regulatory region of the gene, but this event seems rare. On the contrary, disrupting mutations in tumor suppressor sites that tend to accumulate mutations may highlight those regions that are more prone to be targeted to drive the gene loss-of-function. This condition also encompasses those mutations that occur in the last portion of the protein (e.g., nonsense mutations in residue 2400 of NOTCH2, very close to the N-terminal of the aminoacid sequence), which are thus not included in the *Loss-of-function disrupting* category (see previous).

Loss-of-function inframe. The effect of inframe insertions/deletions in tumor suppressors is less clear than that caused by a reading frame shift. Only if they exhibit a high functional impact (according to CADD score) they are considered as a potential loss-of-function event and thus classified as a driver variant. However, we did not observe this in any of the exome-sequenced samples of a 6,792 pan-cancer cohort.

Cluster inframe. Inframe indels that occur within mutational clusters are observed in both oncogenes and tumor suppressors. The former includes some very well known examples, as the inframe indels in the exons 14 and 15 of FLT3. Examples of the latter would be the CDK2NA loss-of-function subsequent to inframe deletions disrupting the gene binding sites, or the TP53 acquisition of dominant negative phenotypes due to the creation of a particular protein fragment subsequent to inframe indels in its 5th exon.

Supplementary Figures

Supplementary Figure 1

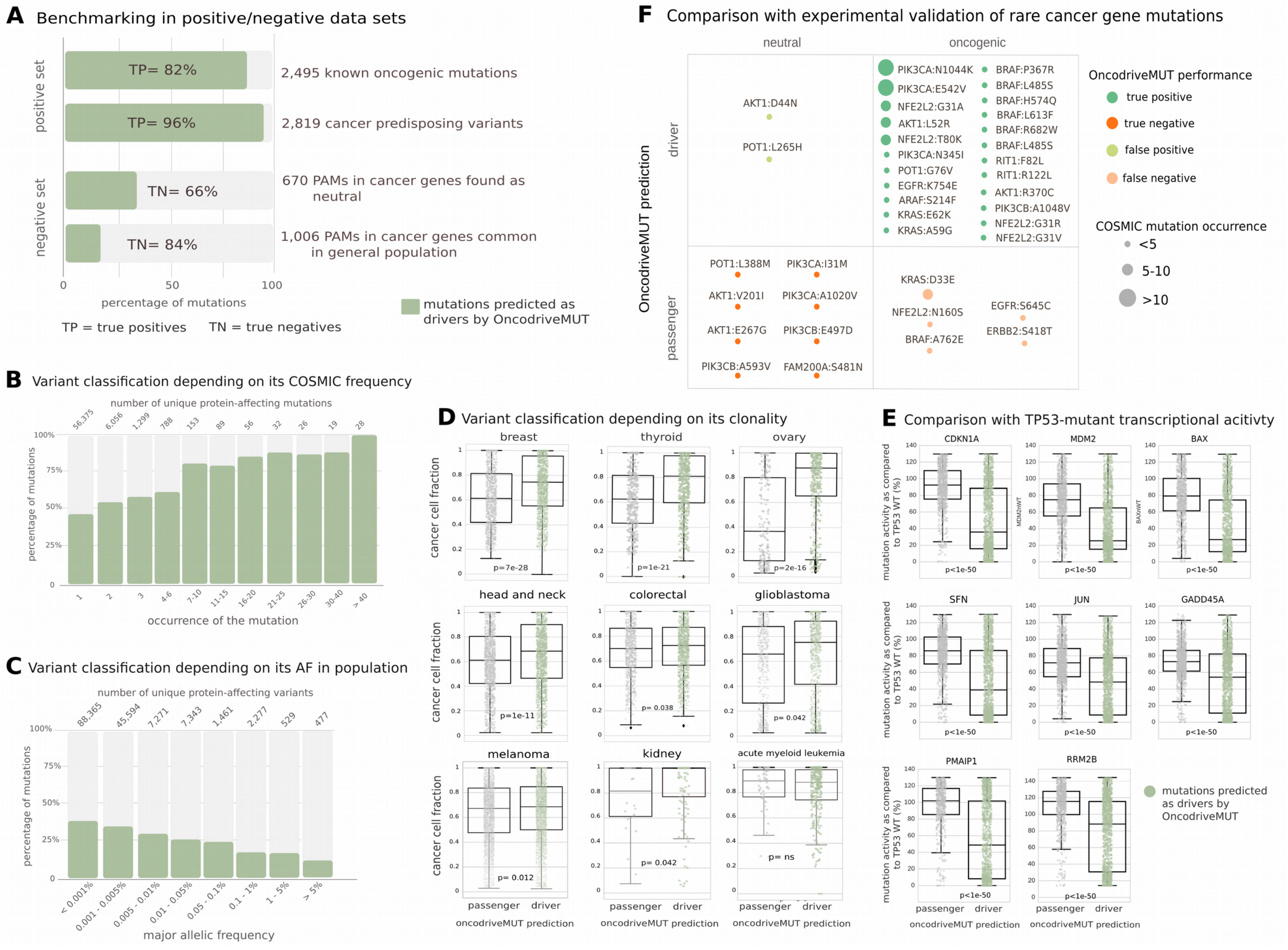

Supplementary Figure 2

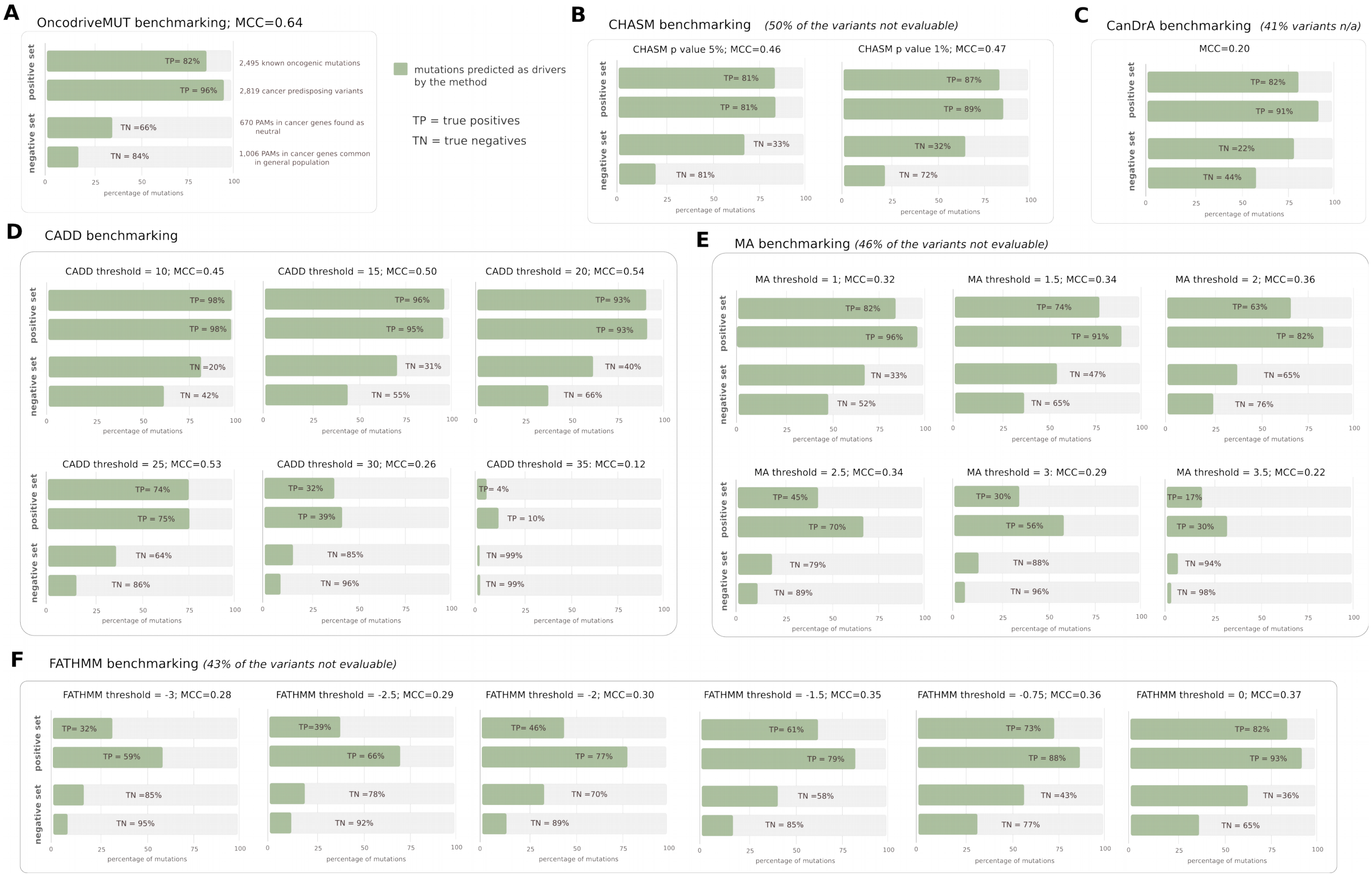

Supplementary Figure 3

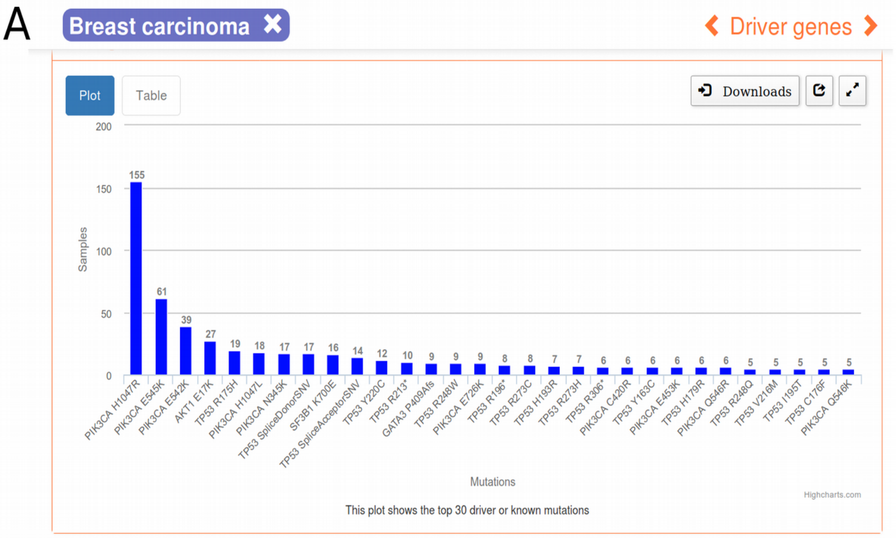

B

Breast carcinoma ✖ PIK3CA ✖ Mut. distribution

Mutations distribution along protein sequence

Plot | Table | Choose a transcript: PIK3CA-001 (1069 aa) | Downloads

Show 10 entries | Search:

Variant Locus	Samples	AA pos	AA change	Consequence	Driver classification
3:178952085:+:A/G	150	1047	H/R	missense_variant	known in: NSCLC;BRCA;COREAD;OV
3:178936091:+:G/A	60	545	E/K	missense_variant	known in: NSCLC;BRCA;COREAD;OV
3:178936082:+:G/A	39	542	E/K	missense_variant	known in: NSCLC;BRCA;COREAD;OV
3:178952085:+:A/T	18	1047	H/L	missense_variant	known in: COREAD;BRCA;NSCLC;OV
3:178921553:+:T/A	17	345	N/K	missense_variant	known in: COREAD;BRCA
3:178938934:+:G/A	9	726	E/K	missense_variant	predicted driver: tier 1
3:178928079:+:G/A	6	453	E/K	missense_variant	known in: CANCER
3:178927980:+:T/C	6	420	C/R	missense_variant	known in: COREAD;BRCA;OV
3:178936095:+:A/G	6	546	Q/R	missense_variant	known in: COREAD;BRCA;OV
3:178952085:+:A/G	5	1047.0	H/R	missense_variant	known in: NSCLC;BRCA;COREAD;OV

Showing 1 to 10 of 72 entries | Previous 1 2 3 4 5 ... 8 Next

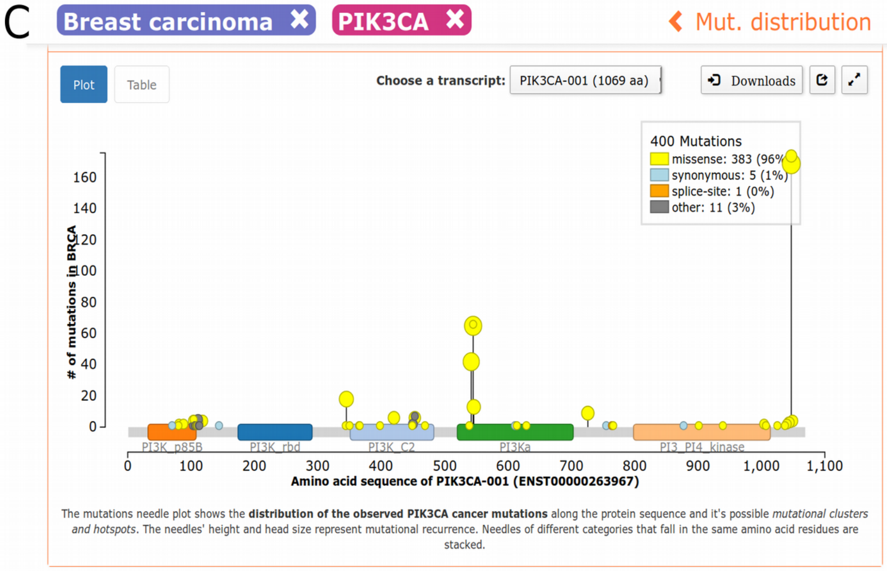

Figure Legends

Suppl. Figure 1

(a) Performance of OncodriveMUT in the classification of validated tumorigenic and neutral protein affecting mutations (PAMs) in cancer genes. Common variants in general population are those with a major allelic frequency >1%.
(b) The fraction of mutations classified as drivers by OncodriveMUT increases with their frequency in cancer (according to COSMIC v76). Note that only PAMs in cancer genes have been included here.
(c) The fraction of germline variants identified as drivers by OncodriveMUT decreases with their prevalence across the general human population (according to ExAC v.0.3.1). Note that only PAMs in cancer genes have been included here.
(d) Cancer cell fraction of PAMs in cancer genes classified as putative drivers or passengers by OncodriveMUT. Mutations in nine tumor types (one in each boxplot) with available data to estimate the clonality have been evaluated. In eight of these cancers, mutations classified as drivers exhibit larger intra-tumor cancer cell fractions than passengers (Mann-Whitney two-sided *p* values are shown).
(e) Biological activity of TP53 missense-mutants classified as putative driver or passenger by OncodriveMUT. The transactivation activity of the TP53 gene carrying each mutation in eight different reporter genes (one in each boxplot) was measured in yeast assays. Each dot represents a different TP53 missense mutant. The value of the y axis represents the activity of the mutant allele relative to the wild-type (i.e. a value below 100% means that the TP53-mutant exhibits a lower transactivation activity than the wild-type allele). Mutations classified as drivers by OncodriveMUT exhibit a lower transactivation activity than passengers across all reporter genes (Mann-Whitney two-sided *p* values are shown).
(f) OncodriveMUT classification of several rarely observed mutations in oncogenes shows a high degree of agreement with the experimental assessment of their tumorigenic effect. Note that none of these mutations are included among those considered for the analysis of panel (A).

Suppl. Figure 2
(a) OncodriveMUT exhibited a Matthew's correlation coefficient (MCC) of 0.64 in separating *bona fide* oncogenic and neutral mutations found in cancer genes and used as benchmarking datasets. Depending on the cutoffs used to state each mutation as oncogenic in other methods with similar purposes, (b) CanDrA produced a MCC of 0.2; **(c)** Cancer-specific High-throughput Annotation of Somatic Mutations (CHASM) produced a MCC ranging between 0.46 and 0.47; **(d)** Combined Annotation Dependent Depletion (CADD) produced a MCC ranging between 0.12 and 0.54; **(e)** MutationAssessor produced a MCC ranging between 0.22 to 0.36; and **(f)** Functional Analysis through Hidden Markov Models (FATHMM) produced a MCC ranging between 0.28 and 0.37. Of note, not all the variants -e.g. indels- can be analyzed by these methods (the percentage of the variants that could not be analysed by each is detailed in the panel legend as appropriate). CanDrA results were retrieved via the Version (Plus) pipeline. Of note, all the variants classified as passenger or drivers by the method were considered regardless of their significance value (calculated as the fraction of mutations that have more extreme scores in the same class in the training data), since the use of any threshold in this value reduced drastically the number of variants that can be evaluated (e.g 5.3% of the variants are classified with a CanDrA significance value lower than 0.05). CADD scores were retrieved via their pipeline v1.3; FATHMM (v2.3), CHASM (v4.0) and Mutation Assessor (release 3) results were retrieved by using the corresponding web sites (http://fathmm.biocompute.org.uk/cancer.html , http://www.cravat.us/, http://mutationassessor.org/r3/). Of note, we used a general configuration for those methods in which the cancer type can be stated as a parameter of the analysis. This is due to the fact that the

cancer type is not annotated for all the variants (specially the negative data set); and even if this information is available, some methods do not take all the cancer types into consideration for their classification.

Suppl. Figure 3

The catalog of driver mutations retrieved by the CGI analysis of a 6,792 tumors pan-cancer cohort is available as a resource at http://www.intogen.org
(a) The results can be browsed at the level of tumor type. In the example, the most frequently gene mutations of breast adenocarcinoma are shown.
(b) The results can be browsed at the level of gene variant, including whether it is a validated oncogenic event (based on the Catalog of Validated Oncogenic Mutations) or whether it is classified as a putative driver *versus* passenger event (based on the OncodriveMUT analysis) otherwise. In the example, the results for the set of PIK3CA mutations observed in breast adenocarcinomas are shown.
(c) The distribution of variants across protein domains can be seen in an interactive graphic. In the example, mutations observed in breast adenocarcinoma tumors across the PIK3CA protein are shown.

Supplementary legend for Figure 3

Cancer acronyms of the tumors gathered by the GENIE project.
The cancer acronyms used in the main Figure 3 are detailed above. Note that tumors were grouped according to the most specific subtype available in the patient information.

RA : renal angiomyolipoma
SCHW : Schwannoma
BLCA : bladder carcinoma
PAAD : pancreas adenocarcinoma
GBM : glioblastoma multiforme
MA : malignant astrocitoma
COREAD : colorectal adenocarcinoma
OV : serous ovarian adenocarcinoma
RCCC : renal clear cell carcinoma
CM : cutaneous melanoma
LIP : liposarcoma
G : glioma
UCEC : uterine corpus endometrioid carcinoma
SOLID : solid tumor
BRCA : breast carcinoma
AML : acute myeloid leukemia
LUAD : lung adenocarcinoma
SCC : squamous cell carcinoma
BCC : basal cell carcinoma
GIST : gastrointestinal stromal cancer
HNSC : head and neck squamous cell carcinoma
HCL : hairy-Cell leukemia
CER : cervix cancer
FRS : female reproductory system cancer
MESO : mesothelioma
LUSC : lung squamous cell carcinoma
BT : billiary tract cancer
TH : thyroid cancer
CH : cholangiocarcinoma
L : lung cancer
NSCLC : non small cell lung carcinoma
LK : leukemia
DBCL : diffuse large B cell lymphoma
THYM : thymic cancer
ESCA : esophageal carcinoma
HNC : head and neck cancer
STAD : stomach adenocarcinoma
PA : pilocytic astrocytoma
ALL : acute lymphoid leukemia
SK : skin cancer
RPC : renal papillary cell

MEN : meningioma
UVM : uveal melanoma
CTCL : cutaneous T-cell lymphoma
PRAD : prostate adenocarcinoma
AS : angiosarcoma
LY : lymphoma
HSEC: Erdheim-Chester histiocytosis
SCLC : small cell lung carcinoma
ES : endocrine system cancer
MERC : Merkel cell carcinoma
MM : multiple myeloma
HC : hepatic carcinoma
MPN : malignant peripheral nerve sheat tumor
THF : thyroid follicular
S : sarcoma
NHLY : non-hodking lymphoma
HLY : hodking lymphoma
CML : chronic myelogenous leukemia
T : testis cancer
B : brain cancer
RHBDS : rhabdomyosarcoma
MDS : myelodisplasic syndrome
CLL : chronic lymphocytic leukemia
DFS : dermatofibrosarcoma
WT : Wilms tumor
M : melanoma
UG: urogenital cancer-related
